# R- and S-Equol have equivalent cytoprotective effects in Friedreich’s Ataxia

**DOI:** 10.1186/2050-6511-13-12

**Published:** 2012-10-22

**Authors:** Timothy E Richardson, James W Simpkins

**Affiliations:** 1 Institute for Aging and Alzheimer’s Disease Research, Department of Pharmacology & Neuroscience, University of North Texas Health Science Center, Fort Worth, TX, 76107, USA; 2 Texas College of Osteopathic Medicine, University of North Texas Health Science Center, Fort Worth, TX, 76107, USA

**Keywords:** Equol, 17β-estradiol, Antioxidants, Friedreich’s Ataxia, Fibroblasts, Neuroprotection

## Abstract

**Background:**

Estradiol (E2) is a very potent cytoprotectant against a wide variety of cellular insults in numerous different cell models, including a Friedreich’s ataxia (FRDA) model. Previously, we demonstrated that estrogen-like compounds are able to prevent cell death in an FRDA model independent of any known estrogen receptor (ER) by reducing reactive oxygen species (ROS) and the detrimental downstream effects of ROS buildup including oxidative damage to proteins and lipids and impaired mitochondrial function.

**Results:**

We have previously demonstrated by western blot that our cell model lacks ERα and expresses only very low levels of ERβ. Using L-buthionine (S,R)-sulfoximine (BSO) to induce oxidative stress in human FRDA fibroblasts, we determine the potency and efficacy of the soy-derived ERβ agonist S-equol and its ERα-preferring enantiomer, R-equol *in vitro* on cell viability and ROS accumulation. Here we demonstrate that these equol biphenolic compounds, while significantly less potent and efficacious than E2, provide statistically similar attenuation of ROS and cytoprotection against a BSO-induced oxidative insult.

**Conclusions:**

These preliminary data demonstrate that estrogen and soy-derived equols could have a beneficial effect in delaying the onset and decreasing the severity of symptoms in FRDA patients by an antioxidant mechanism. In addition, these data confirm that the protection seen previously with E2 was indeed unrelated to ER binding.

## Background

First recognized in 1863 [1], Friedreich’s Ataxia (FRDA) is the most common hereditary form of ataxia characterized by an autosomal recessive GAA trinucleotide repeat in the *FXN* gene, resulting in the absence of frataxin protein [2,3]. The exact function of frataxin is unclear, however it is necessary for iron metabolism within cells, Fe-S cluster assembly in proteins, and maintenance of cellular redox state. Without sufficient levels of frataxin, reactive oxygen species (ROS) begin to accumulate and cells are unable to maintain function of Fe-S cluster proteins essential for mitochondrial respiration leading to mitochondrial dysfunction, insufficient energy production and ultimately cell death, beginning in organs with greater energy requirements and thus more dependent on aerobic ATP production, such as the heart, brain and spinal cord. Symptoms usually begin in the second decade of life and include ataxia, neural hearing and ocular abnormalities, scoliosis, diabetes and cardiomyopathy, which is the most common cause of premature death in FRDA patients [for review see Ref [4].

First detected in humans in 1982 [5], equol is a biphenolic isoflavone metabolized from the soy product daidzein by intestinal flora [6-8] in 14-59% of the human population [9]. Equol is known to act as an antioxidant [10,11], decreases circulating estrogens and androgens [12], inhibits DHT binding to its receptor [13] and decreases risks of prostate [9,11,14] and breast cancer [15]. Separation of racemic equol mixtures shows that S-equol binds with very high affinity to ERβ (K_d_ ~ 0.73 nM), while its enantiomer, R-equol has a far lower affinity for ERβ, instead showing a preference for ERα (K_d_ ~ 15.4 nM), while E2 has a K_d_ ~ 0.05-0.1 nM [16,17]. These enantiomers allow for the discrimination between effects due to antioxidant effects and those due to ERβ activation.

We have previously shown that phenolic estrogens are able to prevent BSO-induced FRDA skin fibroblast death, as well as block the formation of ROS [18], prevent lipid peroxidation, protein damage, depletion of ATP and support the mitochondria and oxidative phosphorylation [19]. In the present study, we provide further evidence that E2 acts by an ERα- and ERβ-independent mechanism. In addition, we demonstrated a lack of ERα and a very low level of ERβ in FRDA fibroblasts by western blot [19]. Here, we show pharmacologically that ERβ is not contributing to this process, as R- and S-equol have statistically equivalent efficacies and potencies, represented here as EC_50_ values. These data indicate that it is the phenolic ring present in the compound structure of equol and E2 and not intrinsic receptor binding ability that is responsible for cytoprotective effects in this FRDA cell model. Although these compounds are substantially less efficacious and potent than compounds previously used [18], this pharmacologic model lends support to the non-receptor mediated, non-genomic antioxidant mechanism of E2.

## Results

### The effects of R- and S-equol on cell viability in BSO-treated FRDA fibroblasts

**Figure 1:**
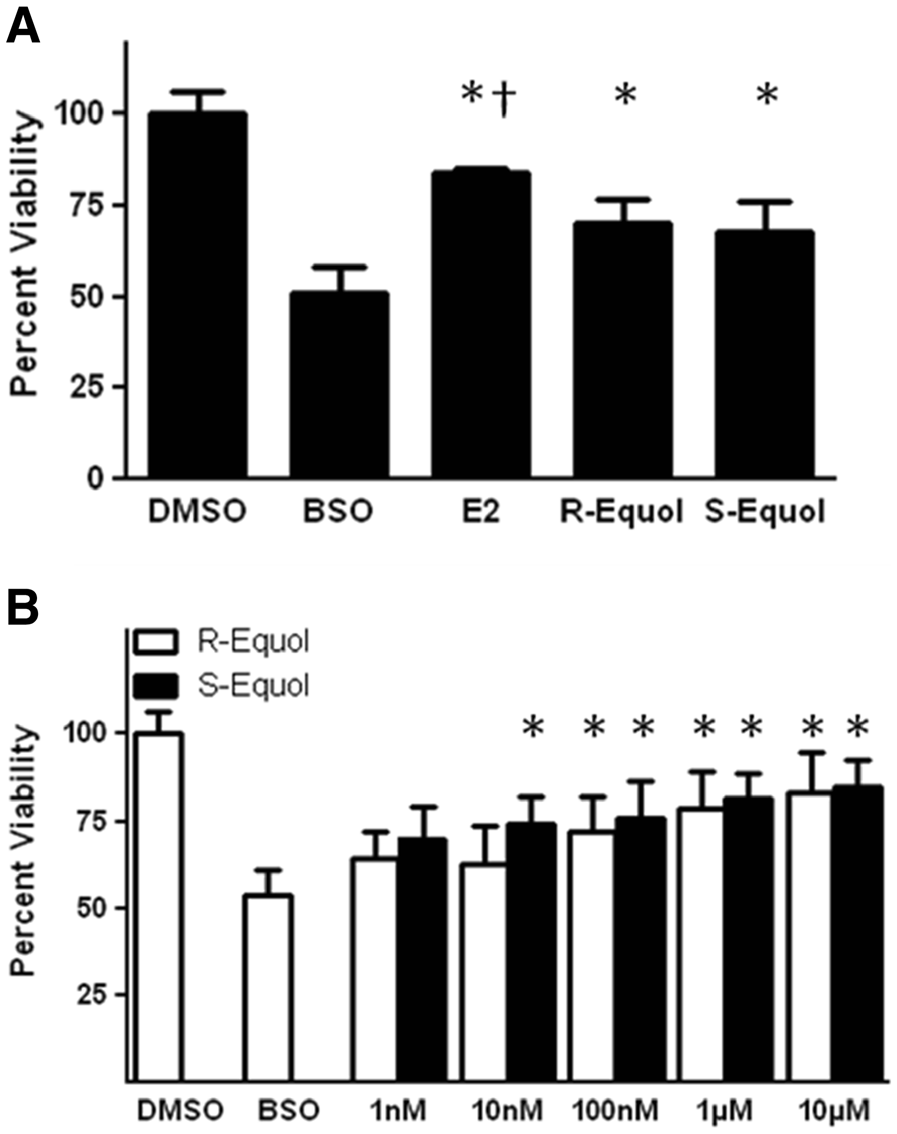
Structures of compounds assessed for protection against BSO toxicity in FRDA fibroblasts.

**Figure 2:**
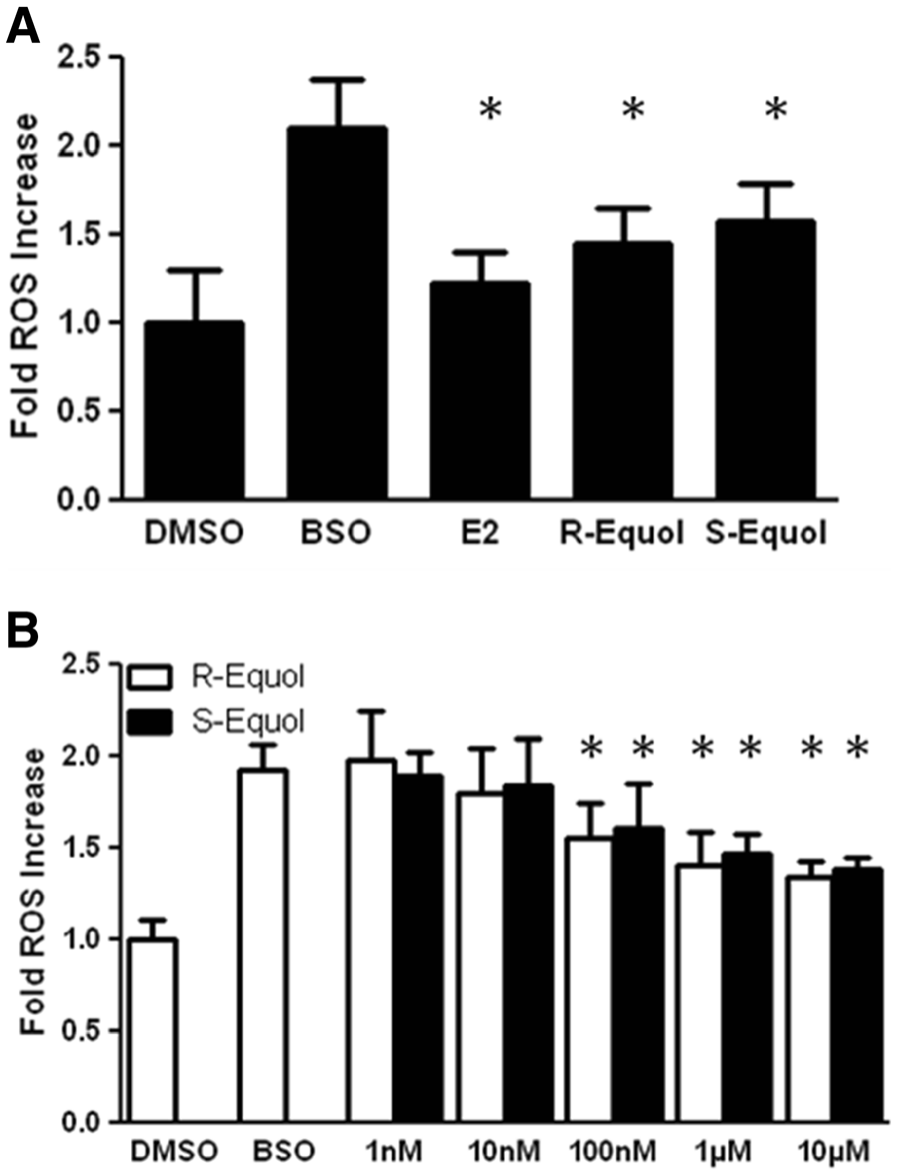
**A.) Effects of 100nM 17β-estradiol, R-equol and S-equol on cell viability in BSO-treated FRDA fibroblasts.B**.) Effects R-equol and S-equol on cell viability in BSO-treated FRDA fibroblasts. Depicted are mean ± SD for n= 8 per group. * indicated p<0.05 versus BSO alone-treated cells. † indicated p<0.05 versus BSO + R- or S-equol.

To determine the effect of R- and S-equol (Figure 1) on cell viability, we first assessed their protective potential compared to 17β-estradiol (E2) at 100nM, a concentration previously shown to be very protective in this cell model [18]. At 100nM, both R- and S-equol provided statistically significant protection compared to the BSO-alone treated group, however the two groups did not differ significantly from each other (Figure 2a). E2 also provided significantly more protection than either of these two compounds (Figure 2a). A dose–response assessment showed that R- and S-equol have almost identical cytoprotective profiles at all concentrations (Figure 2b), and EC_50_ evaluation demonstrated that the two have statistically equivalent EC_50_ values (Table 1), indicating that the cytoprotective effect is not due to stimulation of ERβ. 

**Table 1:** **EC**_**50**_**values for R- and S-equol with respect to cell viability and ROS attenuation**

**Cell Viability**		
**Compound**	**EC50 (nM)**	**Standard Error (nM)**
R-Equol	440.5	21.11
S-Equol	459.9	12.75
**Reactive Oxygen species**		
**Compound**	**EC50 (nM)**	**Standard Error (nM)**
R-Equol	413.9	34.91
S-Equol	439.1	33.77

### The effects of R- and S-equol on BSO-induced reactive oxygen species (ROS) formation

**Figure 3:**
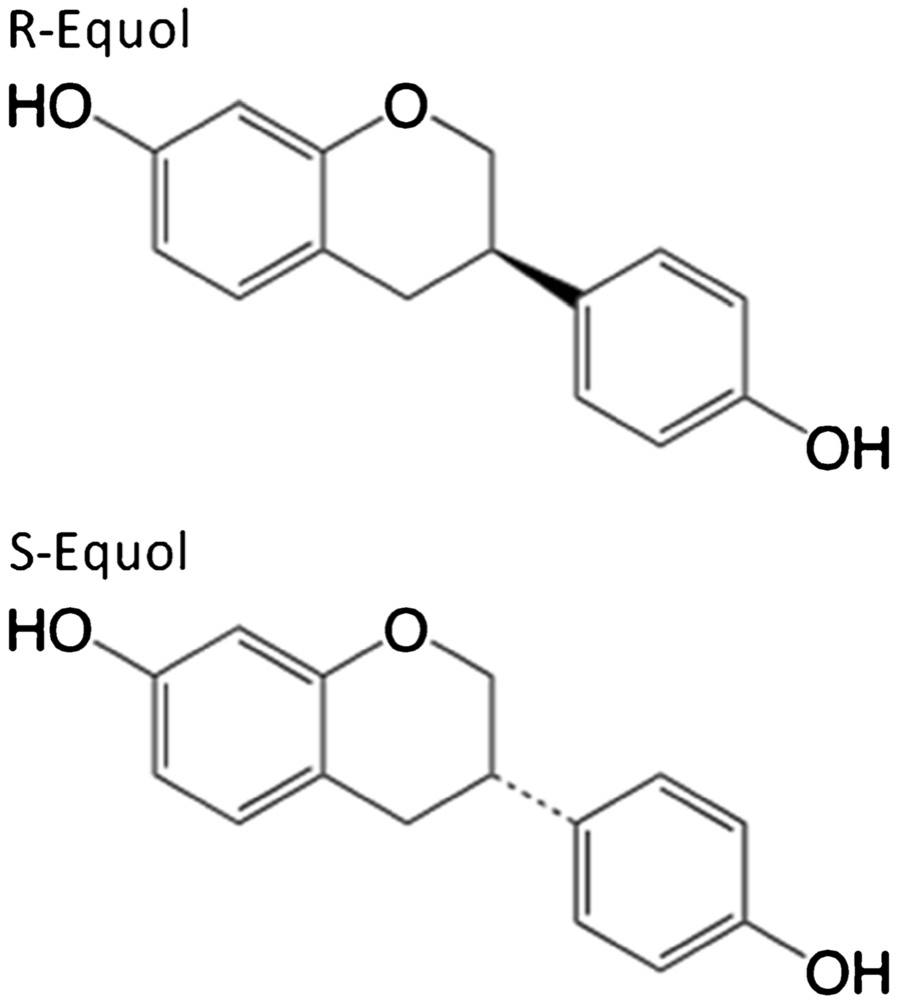
**A.) Effects of 100nM 17β-estradiol, R-equol and S-equol on ROS accumulation in BSO-treated FRDA fibroblasts.B**.) Effects of 100nM R-equol and S-equol on ROS accumulation in BSO-treated FRDA fibroblasts. Depicted are mean ± SD for n= 8 per group. * indicated p<0.05 versus BSO alone-treated cells.

To determine the effects of R- and S-equol on ROS attenuation, these two compounds were again compared to E2 (Figure 3a). BSO induced a 2-fold increase of ROS, which was prevented by 100nM concentrations of E2, R-equol and S-equol. None of these groups differed from each other. In addition, a dose response curve for R- and S-equol shows that there is no significant difference in the ROS attenuation profiles of these two compounds at any concentration (Figure 3b), and the EC_50_ values do not differ significantly (Table 1).

## Discussion

FRDA is the most common of the inherited ataxias world wide, affecting an estimated 1:50,000 to 1:20,000 people [2,4]. With the effective loss of functional frataxin throughout all organ systems, and the resulting ROS proliferation and mitochondrial respiration impairment, cells in organs most dependent on ATP production begin to degenerate [4,20]. This results in the loss of cells in the posterior columns and spinocerebellar tracts of the spinal cord, resulting in tremor and ataxia, as well as lateral and kyphoscoliosis, weakness, speech problems, pes cavitus, an increased incidence of diabetes mellitus and glucose intolerance and cardiac disorders, such as hypertrophic cardiomyopathy with interstitial fibrosis [4]. Disease onset and severity is variable depending on the number of GAA trinucleotide repeats present in the first intron of the *FXN* gene, although this alone is not able to account for the full course of the disease process [21]. There is little difference between males and females in terms of disease onset, progress and severity as this is inherited in an autosomal recessive manner and symptoms begin in the first 2 decades of life, before hormone level changes in puberty [22].

Estrogen and non-feminizing estrogens have been shown to be potently cytoprotective in many different cell and animal models of disease states [23,24], including a FRDA cell model [18]. Previous observations have demonstrated that antioxidants, especially mitochondrially targeted antioxidants [25,26], including estrogen receptor agonists and non-feminizing estrogens [18] are protective against FRDA. These effects have been shown to be ER independent and are instead based in the antioxidant properties of phenolic estrogens [27,28].

Estrogens exert both genomic and non-genomic effects on redox status of cells for reviews see [29-34]. Unfortunately, no studies on the genomic effects of estrogens has been published using FRDA cells, but we have reported that these cells respond to estrogens even in the presence of a pan-estrogen receptor inhibitors, ICI 182780 [18], have no detectable ERα and low levels of ERβ [19], and exhibit these effects at concentrations in excess of the ED_50_ for 17β-estradiol [18]. Nonetheless, genomic effects of estrogens on antioxidant enzymes have been reported, which could contribute to estrogen’s antioxidant effects. For example, tamoxifen is reported to up-regulated the quinine reductase, NQO1 [29], and estrogens up-regulate expression of peroxidase-1 and MnSOD [30]. In contrast, Pajovic and Saicic [31] have reported that MnSOD, glutathione peroxidase, glutathione-S-transferase and glutathione reductase are decreased by estradiol, whereas catalase is increased. The extent to which the non-genomic effects of estrogens influence these paradoxical decreases in antioxidant enzyme expression is not known.

Estrogens are highly lipid soluble (the logarithm of the octanol/water partition coefficient, log *P*, is 3.35) and largely reside in the membrane component of cells [35] where they are ideally suited to affect oxidation of unsaturated bonds in phospholipids. Indeed, estrogens appear to intercalate into the membrane with their phenolic A ring situated near the site of lipid peroxidation [36]. We reasoned that estrogens may interrupt lipid peroxidation chain reactions via oxidation in a manner that could be redox-cycled back to the parent estrogen, using a plentiful and regenerable source of cellular reducing potential, such as glutathione or NADPH. We discovered that estrogens were converted via hydroxyl radical exposure to a quinol product that was, in turn, enzymatically reduced back to the parent estrogen in the presence of NAD(P)H as a co-factor [27,28]. This estrogen redox cycle is operative in the central nervous system [27] where it serves, together with the “classical” antioxidant mechanism for phenolic compounds, as a defense mechanism against ROS.

Equol is a naturally derived biphenolic (Figure 1) product of soy digestion in a substantial percentage of the American population [5]. It is created by intestinal flora as a racemic mixture of the R- and S-forms, with the S-form being very selective for ERβ, the only ER present in FRDA fibroblasts [19] while the R-form is only a very weak agonist at this receptor [16,17]. Our results indicate that, while not as potent or efficacious as E2 (Figure 2a and 3a) [18], the R- and S-forms of equol are equally effective in attenuating ROS (Figure 3b, Table 1) and preventing cell death (Figure 2b, Table 1). These data indicate that equol, specifically the non-feminizing R-equol, could potentially be used to prevent or delay cell death and pathologic symptoms in FRDA and supports our previous hypothesis that estrogen-like compounds are acting in a manner unrelated to any known ER [18,19]. 

## Conclusions

Because the biphenolic compounds R- and S-equol have statistically equal cytoprotective profiles despite extremely different ERβ binding profiles, these data confirm that ERβ is not involved in the protective effects of E2 seen previously in this FRDA fibroblast model [18,19]. Furthermore, this study demonstrates that estrogen and soy-derived equols are effective at reducing ROS and improving cell viability in FRDA fibroblasts and shows that naturally derived soy estrogens could have a beneficial effect in delaying the onset and decreasing the severity of symptoms in FRDA patients by an antioxidant mechanism. These data add more weight to the neuroprotective hypothesis of estrogen and provide evidence that E2 and other phenol ring-containing estrogens should be considered as candidate drugs for the treatment and prevention of the symptoms of FRDA.

## Methods

### Cell culture

Fibroblasts from a 30 year old Friedreich’s Ataxia (FRDA) patient (Coriell Institute, Camden NJ, USA) were maintained in Dulbecco’s Modified Eagle Medium (DMEM; ThermoScientific, Waltham, MA, USA) with 10% charcoal-stripped fetal bovine serum (CSFBS; ThermoScientific, Waltham, MA, USA), 1% GlutaMAX (ThermoScientific, Waltham, MA, USA) and 1% penicillin-streptomycin (Invitrogen, Carlsbad, CA, USA) at 37°C in 90% humidity and 5% CO_2_. At the time of treatment, the FRDA fibroblast media was changed to phenol red- and sodium pyruvate-free DMEM (ThermoScientific, Waltham, MA, USA) and 1% penicillin-streptomycin. Experiments were conducted using cell passages 15–19.

### Chemicals & reagents

17β-Estradiol (E2) was acquired from Steraloids, Inc. (Newport, RI, USA). L-buthionine (S,R)-sulfoximine (BSO) was obtained from Sigma-Aldrich (St Louis, MO, USA). R- and S- Equol were obtained from the laboratory of Dr Robert J Handa at The University of Arizona.

### Treatment paradigm

FRDA fibroblasts were removed from culture with 0.25% Trypsin-EDTA (Invitrogen, Carlsbad, CA, USA) and plated on 96-well plates at a density of 3000 cells per well in DMEM with 10% CSFBS, 1% GlutaMAX and 1% penicillin-streptomycin. After 24 hours the media was removed and replaced with phenol red- and sodium pyruvate-free DMEM with 1% penicillin-streptomycin. The cells were then treated for 12 to 48 hours with either dimethyl sulfoxide vehicle control (DMSO; Sigma-Aldrich, St Louis, MO, USA) or 1mM BSO in the presence of E2, R-equol or S-equol ((3S)-3-(4-Hydroxyphenyl)-7-chromanol). This duration of exposure was chosen based on our observation of BSO-induced enhacement of ROS at 12 hours and cell death at 48 hours [18].

### Calcein AM cell viability assay

Cells were plated on a 96-well plate at a density of 5,000 cells per well, then treated with vehicle or 1mM BSO. After 48 hours of BSO treatment, the media was removed, and 1 μg/mL Calcein AM (CalBiochem, San Diego, CA, USA) in phosphate buffer pH 7.2 (PBS; Fisher Scientific, Pittsburg, PA, USA) was added to each well and the plate was incubated for 10 minutes at 37°C. Cell viability was determined with a Tecan Infinite M200 (Tecan Systems, Inc., San Jose, CA) plate reader with an excitation of 490nm and emission of 520nm at 48 hours.

### Reactive oxygen species assay

After 12 hours of treatment the media was removed from each well of the 96-well plate, and 100μL of a 1μM 2’,7’-Dichlorodihydrofluorescein diacetate (DCFDA; AnaSpec Inc., Fremont, CA, USA) in PBS was added to each well. The plates were returned to a 37°C incubator for 20 minutes, then each well was washed three times with PBS and the resulting reaction was read on a Tecan Infinite M200 plate reader with an absorbance of 495 nm and an emission of 529 nm.

### Data and statistics

All data are displayed as mean ± 1 standard deviation. These data were analyzed using the ANOVA against an alpha level of 0.05. All bar graphs were made using GraphPad Prism 5 and EC_50_ calculations were made with GraphPad Prism 5. For all groups, n=8 wells and experiments were repeated three times to ensure consistency.

## Abbreviations

BSO: L-buthionine (S,R)-sulfoximine; FRDA: Friedreich’s Ataxia; E2: 17β-Estradiol; ROS: Reactive oxygen species; ER: Estrogen receptor; ERα: Estrogen receptor α; ERβ: Estrogen receptor β.

## Competing interests

The authors declare that they have no competing interests.

## Authors’ contributions

TER carried out the experiments, performed statistical analysis and wrote the initial draft of the manuscript. JWS revised and approved the final manuscript. All authors were involved with the conception and design of the studies. Both authors read and approved the final manuscript.

## Pre-publication history

The pre-publication history for this paper can be accessed here:

http://www.biomedcentral.com/2050-6511/13/12/prepub

## References

[B1] Friedreich N Uber degenerative Atrophie der spinalen Hinterstrange Arch Pathol Anat Phys Klin Med 1863 26 391 419

[B2] Harding AE Classification of the hereditary ataxias and paraplegias Lancet 1983 1 1151 1155 6133167 10.1016/s0140-6736(83)92879-9

[B3] Campuzano V Montermini L Moltò MD Pianese L Cossée M Cavalcanti F Monros E Rodius F Duclos F Monticelli A Zara F Cañizares J Koutnikova H Bidichandani SI Gellera C Brice A Trouillas P De Michele G Filla A De Frutos R Palau F Patel PI Di Donato S Mandel JL Cocazza S Koenig M Pandolfo M Friedreich’s ataxia: autosomal recessive disease caused by an intronic GAA triplet repeat expansion Science 1996 271 1423 1427 10.1126/science.271.5254.1423 8596916

[B4] Santos R Lefevre S Sliwa D Seguin A Camadro JM Lesuisse E Friedreich ataxia: molecular mechanisms, redox considerations and therapeutic opportunities Antioxid Redox Signal 2010 13 651 690 10.1089/ars.2009.3015 20156111 PMC2924788

[B5] Axelson M Kirk DN Farrant RD Cooley G Lawson AM Setchell KD The identification of the weak oestrogen equol [7-hydroxy-3-(4’-hydroxyphenyl)chroman] in human urine Biochem J 1982 201 353 357 7082293 10.1042/bj2010353 PMC1163650

[B6] Wang XL Hur HG Lee JH Kim KT Kim SI Enantioselective synthesis of S-equol from dihyrodaidzein by a newly isolated anaerobic human intestinal bacterium Appl Environ Microbiol 2005 71 214 219 10.1128/AEM.71.1.214-219.2005 15640190 PMC544246

[B7] Price KR Fenwick GR Naturally occurring oestrogens in foods – a review Food Addit Contam 1985 2 73 106 10.1080/02652038509373531 4018320

[B8] Kelly GE Nelson C Waring MA Joannou GE Reeder AY Metabolites of dietary (soya) isoflavones in human urine Clin Chim Acta 1993 223 9 22 10.1016/0009-8981(93)90058-C 8143372

[B9] Akaza H Miyanaga N Takashima N Naito S Hirao Y Tsukamoto T Fujioka T Mori M Kim WJ Song JM Pantuck AJ Comparisons of percent equol producers between prostate cancer patients and controls: case-controlled studies of isoflavones in Japanese, Korean and American residents Jpn J Clin Oncol 2004 34 86 89 10.1093/jjco/hyh015 15067102

[B10] Pereboom D Gilaberte Y Sinues B Escanero J Alda JO Antioxidant intracellular activity of genistein and equol J Med Food 1999 2 253 256 10.1089/jmf.1999.2.253 19281394

[B11] Mitchell JH Gardner PT McPhail DB Morrice PC Collins AR Duthie GG Antioxidant efficacy of phytoestrogens in chemical and biological model systems Arch Biochem Biophys 1998 360 142 148 10.1006/abbi.1998.0951 9826439

[B12] Duncan AM Merz-Demlow BE Xu X Phipps WR Kurzer MS Premenopausal equol excretors show plasma hormone profiles associated with lowered risk of breast cancer Cancer Epidemiol Biomarkers Prev 2000 9 581 586 10868692

[B13] Lund TD Munson DJ Haldy ME Setchell KD Lephart ED Handa RJ Equol is a novel anti-androgen that inhibits prostate growth and hormone feedback Biol Reprod 2004 70 1188 1195 14681200 10.1095/biolreprod.103.023713

[B14] Mitchell JH Duthie SJ Collins AR Effects of phytoestrogens on growth and DNA integrity in human prostate tumor cell lines: PC-3 and LNCaP Nutr Cancer 2000 38 223 228 10.1207/S15327914NC382_12 11525601

[B15] Frankenfeld CL McTiernan A Aiello EJ Thomas WK LaCroix K Schramm J Schwartz SM Holt VL Lampe JW Mammographic density in relation to daidzein-metabolizing phenotypes in overweight, postmenopausal women Cancer Epidemiol Biomarkers Prev 2004 13 1156 1162 15247126

[B16] Muthyala RS Ju YH Sheng S Williams LD Doerge DR Katzenellenbogen BS Helferich WG Katzenellenbogen JA Equol, a natural estrogenic metabolite from soy isoflavones: convenient preparation and resolution of R- and S-equols and their differing binding and biological activity through estrogen receptors alpha and beta Bioorg Med Chem 2004 12 1559 1567 10.1016/j.bmc.2003.11.035 15018930

[B17] Setchell KD Clerici C Lephart ED Cole SJ Heenan C Castellani D Wolfe BE Nechemias-Zimmer L Brown NM Lund TD Handa RJ Heubi JE S-Equol, a potent ligand for estrogen receptor {beta}, is the exclusive enantiomeric form of the soy isoflavone metabolite produced by human intestinal bacterial flora Am J Clin Nutr 2005 81 1072 1079 15883431 10.1093/ajcn/81.5.1072

[B18] Richardson TE Yang SH Wen Y Simpkins JW Estrogen protection in Friedreich’s ataxia skin fibroblasts Endocrinology 2011 152 2742 2749 10.1210/en.2011-0184 21540287 PMC3115615

[B19] Richardson TE Yu AE Wen Y Yang SH Simpkins JW Estrogen prevents oxidative damage to the mitochondria in Friedreich’s ataxia skin fibroblasts PLoS One 2012 7 e13600 10.1371/journal.pone.0034600 PMC3318005 22509330

[B20] Marmolino D Friedreich’s ataxia: past, present and future Brain Res Rev 2011 67 311 330 10.1016/j.brainresrev.2011.04.001 21550666

[B21] Klopstock T Chahrokh-Zadeh S Holinski-Feder E Meindl A Gasser T Pongratz D Müller-Felber W Markedly different course of Friedreich’s ataxia in sib pairs with similar GAA repeat expansions in the frataxin gene Acta Neuropathol 1999 97 139 142 10.1007/s004010050966 9928824

[B22] Leone M Brignolio F Rosso MG Curtoni ES Moroni A Tribolo A Schiffer D Friedreich’s ataxia: a descriptive epidemiological study in an Italian population Clin Genet 1990 38 161 169 2225525 10.1111/j.1399-0004.1990.tb03566.x

[B23] Simpkins JW Green PS Gridley KE Singh M de Fiebre NC G Rajakumar G Role of estrogen replacement therapy in memory enhancement and the prevention of neuronal loss associated with Alzheimer's disease Am J Medicine 1997 103 195 255 10.1016/s0002-9343(97)00260-x 9344403

[B24] Behl C Oestrogen as a neuroprotective hormone Nat Rev Neurosci 2002 3 433 442 12042878 10.1038/nrn846

[B25] Jauslin ML Wirth T Meier T Shoumacher F A cellular model for Friedreich Ataxia reveals small-molecule glutathione peroxidase mimetics as novel treatment strategy Hum Mol Genet 2002 11 3055 63 10.1093/hmg/11.24.3055 12417527

[B26] Jauslin ML Meier T Smith RA Murphy MP Mitochondria-targeted antioxidants protect Friedreich Ataxia fibroblasts from endogenous oxidative stress more effectively than untargeted antioxidants FASEB J 2003 17 1972 4 12923074 10.1096/fj.03-0240fje

[B27] Prokai L Prokai-Tatrai K Perjesi P Simpkins JW Mechanistic insights into the direct antioxidant effects of estrogens Drug Dev Res 2006 66 118 125

[B28] Prokai L Prokai-Tatrai K Perjesi P Zharikova AD Perez EJ Liu R Simpkins JW Quinol-based cyclic antioxidant mechanism in estrogen neuroprotection Proc Natl Acad Sci USA 2003 100 11741 11746 10.1073/pnas.2032621100 14504383 PMC208828

[B29] Montano MM Bianco NR Deng H Wittmann BM Chaplin LC Katzenellenbogen BS Estrogen receptor regulation of quinone reductase in breast cancer: implications for estrogen-induced breast tumor growth and therapeutic uses of tamoxifen Front Biosci 2005 10 1440 1461 10.2741/1630 15769636

[B30] Viña J Sastre J Pallardó FV Gambini J Borrás C Role of mitochondrial oxidative stress to explain the different longevity between genders: protective effect of estrogens Free Radic Res 2006 40 1359 1365 10.1080/10715760600952851 17090425

[B31] Pajović SB Saicić ZS Modulation of antioxidant enzyme activities by sexual steroid hormones Physiol Res 2008 57 801 811 18052675 10.33549/physiolres.931377

[B32] Simpkins JW Dykens JA Mitochondrial mechanisms of estrogen neuroprotection Brain Res Rev 2008 57 421 430 10.1016/j.brainresrev.2007.04.007 17512984

[B33] Duckles SP Miller VM Hormonal modulation of endothelial NO production Pflugers Arch 2010 459 841 851 10.1007/s00424-010-0797-1 20213497 PMC2865573

[B34] White RE Gerrity R Barman SA Han G Estrogen and oxidative stress: a novel mechanism that may increase the risk for cardiovascular disease in women Steroids 2010 75 788 793 10.1016/j.steroids.2009.12.007 20060403 PMC2891201

[B35] Liang Y Belford S Tang F Prokai L Simpkins JW Hughes JA Membrane fluidity effects of estratrienes Brain Res Bull 2001 54 661 668 10.1016/S0361-9230(01)00483-X 11403993

[B36] Cegelski L Rice CV O'Connor RD Caruano AL Tochtrop GP Cai ZY Covey DF Schaefer J Mapping the locations of estradiol and potent neuroprotective analogues in phospholipid bilayers by REDOR NMR Drug Dev Res 2006 66 93 102

